# Social Media Use and Emotional Well-Being: The Role of Physical Activity

**DOI:** 10.1093/geroni/igab046.3345

**Published:** 2021-12-17

**Authors:** Xin Yao Lin, Margie Lachman

**Affiliations:** 1 Brandeis University, Waltham, Massachusetts, United States; 2 Brandeis University, Brandeis University, Massachusetts, United States

## Abstract

There are mixed findings as to whether social media use (SMU) is positively or negatively related to well-being (positive/negative affect), and this relationship varies by age. The current study seeks to further explore this relationship by examining physical activity (PA) as a potential mediator at both a within (intraindividual) and between-person (interindividual) level across adulthood. The data are from the Midlife in the United States Refresher eight-day daily diary study (N=782, ages 25-75) with self-reported frequency of SMU, PA, and well-being (positive/negative affect). Multilevel structural equation modeling simultaneously tested how the relationships between the variables differed at both the between- and within-person levels. Between-person results showed that across the week, those who reported less SMU reported engaging in more PA, and more PA was associated with more positive affect. PA significantly mediated the relationship between SMU and positive affect for midlife and older adults, but not for younger adults. Effects for negative affect were not significant. Within-person results indicated that days with more PA were associated with more positive affect; however, PA did not mediate the relationship between SMU and positive or negative affect. These findings suggest the benefits of engaging in PA on one’s positive emotional well-being at both the between- and within-person levels. However, for midlife and older adults, more SMU across the week may take away time from engaging in PA, which in turn lowers their positive affect. Implications of the effects of SMU on PA and well-being across adulthood are discussed.

